# Estimation of pre-Fontan pulmonary vascular resistance in children with single ventricle heart disease at the Glenn stage: a multicenter study

**DOI:** 10.3389/fped.2025.1698653

**Published:** 2025-11-05

**Authors:** Sebastian Laudenschlager, Dhaval Chauhan, Nita Ray Chaudhuri, Christopher E. Mascio, Jai P. Udassi, Benjamin S. Frank, Jennifer Romanowicz, Yue-Hin Loke, Vitaly O. Kheyfets, Mehdi Hedjazi Moghari

**Affiliations:** ^1^ Department of Pediatrics, West Virginia University and West Virginia University Medicine Children’s Hospital, Morgantown, WV, United States; ^2^ Department of Cardiology, University of Colorado and Children’s Hospital Colorado, Aurora, CO, United States; ^3^ Department of Cardiology, George Washington University and Children’s National Hospital, Washington, DC, United States; ^4^ Cardiovascular Pulmonary Research Laboratories, Division of Pulmonary Sciences and Critical Care Medicine, Department of Medicine, University of Colorado Anschutz Medical Campus, Aurora, CO, United States; ^5^ Division of Pediatrics-Critical Care, Department of Pediatrics, University of Colorado Anschutz Medical Campus, Aurora, CO, United States; ^6^ Department of Biomedical Informatics, University of Colorado Anschutz Medical Campus, Aurora, CO, United States

**Keywords:** pulmonary vascular resistance, Fontan surgery, computational fluid dynamics, cardiovascular magnetic resonance, catheterization

## Abstract

The Fontan procedure, employed in the management of children with single ventricle congenital heart disease, continues to present long-term complications. Notably, certain complications associated with this procedure are linked to imbalances in the distribution of hepatic blood flow. One promising strategy to address this challenge involves employing a digital twin to simulate diverse Fontan configurations. The objective is to identify an optimal design that ensures balanced hepatic blood flow and minimizes power losses. However, successful implementation depends on accurate, patient-specific estimates of pulmonary vascular resistance (PVR) for each lung at the pre-Fontan (Glenn) stage. In clinical practice, only the total PVR is typically measured, via catheterization using the Fick principle, but individual lung resistances can be derived by combining pressure data from catheterization (Cath) with flow data from cardiac magnetic resonance imaging (CMR). Still, notable discrepancies exist: Fick-based total PVR often differs significantly from Cath-CMR-based PVR due to differences in flow quantification, and neither method can distinguish between proximal and distal resistances within the Glenn pathway. An alternative method for estimating PVR was previously developed using a computational fluid dynamics (CFD) optimization framework. This method demonstrated a favorable correlation with PVR estimates derived from Cath-CMR, although it was not directly compared to clinical PVR values derived using the Fick principle. In this study, we compare three methods for calculating PVR, namely Fick-based, Cath-CMR-based, and CFD-based, using patient data from three independent institutions. Our results show that Fick-based PVR values are, on average, significantly lower than those obtained via the Cath-CMR and CFD methods. The CFD-based total PVR estimates show good agreement with the total Cath-CMR-based PVR. However, the elevated left proximal resistance present in the CFD method leads to a significant underestimation of the left lung resistance by the Cath-CMR method. This underscores the significance of incorporating proximal resistance in PVR estimation and supports the potential utility of the CFD-based method for preoperative planning in single ventricle patients.

## 1 Introduction

Single ventricle congenital heart disease is typically managed through a three-stage surgical palliation designed to separate the systemic and pulmonary circulations, thereby bypassing the underdeveloped or nonfunctional ventricle (1). This staged approach reduces the mixing of oxygenated and deoxygenated blood, resulting in improved systemic oxygenation. The surgical stages of palliation consist of: (1) stage 1 palliation or the Norwood procedure, (2) the bidirectional Glenn or hemi-Fontan procedure, and (3) the Fontan completion. Following complete palliation, a total cavopulmonary connection (TCPC) is established in which the superior and inferior vena cavae (SVC and IVC) are directly anastomosed to the left and right pulmonary arteries (LPA and RPA). This anatomical configuration enables passive, non-pulsatile pulmonary perfusion, bypassing the need for a subpulmonary ventricle  (2).

While this staged palliation has significantly improved survival, long-term complications remain prevalent  (3). Many of these are linked to the suboptimal hemodynamic performance of the TCPC  (4, 5), including elevated power loss, which contributes to diminished exercise tolerance (6), and the development of pulmonary arteriovenous malformations (7, 8).

One strategy to optimize post-Fontan hemodynamics involves the use of patient-specific computational fluid dynamics (CFD) models to simulate surgical outcomes and predict the effects of alternative TCPC geometries (9, 10). A critical input to such simulations is an accurate estimate of pulmonary vascular resistance (PVR) in each lung prior to Fontan completion. However, in clinical practice, individual lung PVRs are rarely measured; only total PVR is typically derived during cardiac catheterization (Cath), using pressure measurements and pulmonary flow ($Q_{p}$) calculated via the Fick principle  (11).

Unfortunately, Fick-based estimates of pulmonary flow have been shown to be inconsistent and less reliable than measurements obtained from cardiac magnetic resonance imaging (CMR), particularly in single ventricle patients  (12, 13). As a result, Fick-derived total PVR values may lack accuracy. One aim of this study is to quantify the discrepancy between Fick-based and Cath-CMR-based PVR measurements using patient data from three independent institutions. Another limitation of the Fick-based method for estimating PVR is its inability to provide separate left and right lung resistances, as it only calculates the total lung PVR.

While Cath pressure data combined with CMR flow can be used to estimate individual lung PVRs, this method does not allow for differentiation between proximal and distal resistances along the Glenn pathway. To address this limitation, a CFD-based optimization framework was previously developed to estimate lung-specific PVRs, including proximal resistance components, at the stage 2 (Glenn) physiology  (14). However, this earlier study was limited to a small patient cohort from a single institution.

In this manuscript, we expand upon this prior work by applying the PVR estimation algorithm to a larger, multi-institutional dataset. Our goals are twofold: first, to rigorously compare Fick, Cath-CMR, and CFD-based PVR measurements across three centers, and second, to highlight the importance of the proximal resistances found through the CFD-based PVR estimation framework by benchmarking it against the PVR values derived from the Cath-CMR method.

## 2 Methods

### 2.1 Patient demographic data

Clinical data for this three-center study were retrospectively collected and anonymized from three medical institutions over a ten-year period, with approval from the Institutional Review Board (IRB) of each participating center. Due to the retrospective nature of the study, the requirement for informed consent was waived. Data were accessed and analyzed between January 2023 and May 2025. The dataset comprises CMR and Cath data from 42 pediatric patients (28 males, 14 females; age range: 2–10 years; mean age 3.85 ± 1.49 years) with Glenn physiology (39 bidirectional Glenn, 3 hemi-Fontan), distributed across the three centers as follows:

• Center 1: 10 males, 6 females; age range: 2–5 years; 13 bidirectional Glenn, 3 hemi-Fontan
• Center 2: 14 males, 6 females; age range: 2–6 years; 20 bidirectional Glenn, 0 hemi-Fontan
• Center 3: 4 males, 2 females; age range: 2–10 years; 6 bidirectional Glenn, 0 hemi-Fontan

CMR imaging was used to construct three-dimensional anatomical models for CFD simulations and to quantify time-averaged pulmonary blood flow in the SVC, LPA, and RPA, as previously described (14). Cath data included mean pressure measurements at the SVC, LPA, RPA, and left atrium (LA). All but four patients had their CMR and CATH examinations within the same week. Of the remaining four patients, two had their examinations within a month, one half a year apart, and one a year apart.

### 2.2 Pulmonary vascular resistance

PVR was calculated using three methods: the Fick principle, Cath-CMR-based estimation, and CFD-based modeling. These calculations were conducted for all 42 patients and compared across the three participating centers. All PVR values are reported as body surface area (BSA)-indexed Wood units (iWU or WU·m²).

#### 2.2.1 Estimation of total PVR using the Fick principle

Total PVR, denoted as $R_{total}$, is calculated exclusively from data measured by a Cath exam, using a hydraulic analogue of Ohm's law:

(1) $$R_{total}=\frac{\Delta P_{transpulmonary}}{Q_{p}}=\frac{mPAP-PAWP}{Q_{p}},$$

where mPAP is the mean pulmonary artery pressure, calculated as the average of LPA and RPA pressure measurements, PAWP is the pulmonary artery wedge pressure (an estimate of LA pressure), and $Q_{p}$ is the pulmonary blood flow. Pulmonary blood flow is determined using the Fick principle, which defines flow as the ratio of oxygen consumption ($VO_{2}$) to the arteriovenous oxygen content difference across the pulmonary circulation, which are obtained through Cath. In clinical practice, direct measurement of $VO_{2}$ via indirect calorimetry is considered the gold standard. However, this method is often impractical, particularly in pediatric settings or during Cath-based procedures (15). As a result, $VO_{2}$ is often estimated using predictive equations based on age, body weight, and occasionally heart rate which has limitations in patients with Glenn vasculature (16).

#### 2.2.2 PVR estimation using pressure measurements from Cath and flow measurements from CMR

Individual left and right lung resistances ($R_{LPA}$ and $R_{RPA}$) can be calculated directly from clinical measurements, specifically using pressure data from Cath and flow data from CMR:

(2) $$R_{LPA}\;=\frac{P_{LPA}-P_{LA}}{Q_{LPA}},\;\;R_{RPA}\;=\frac{P_{RPA}-P_{LA}}{Q_{RPA}},$$

where $Q_{LPA}$ and $Q_{RPA}$ represent the flow rates through the LPA and RPA, respectively, as measured by CMR. The corresponding resistances, $R_{LPA}$ and $R_{RPA}$ can then be used to compute the total PVR, equivalent to the LPA and RPA resistance in parallel and analogous to the value obtained using the Fick principle:

(3) $$R_{total}=\frac{R_{LPA}\;\cdot \;R_{RPA}\;}{R_{LPA}\;+\;R_{RPA}\;}.$$

#### 2.2.3 Estimation of PVR using CFD

The individual PVR can also be estimated using a CFD-based optimization algorithm that adjusts the LPA and RPA resistances to minimize the error between simulated and clinical flow and pressure data. This algorithm, implemented using the SimVascular (17) software package, produces separately identifiable distal resistances ($R_{LPA}$ and $R_{RPA}$), as well as proximal resistances ($r_{SVC}$, $r_{LPA}$, and $r_{RPA}$) corresponding to the SVC, LPA, and RPA, respectively.

**Figure 1:**
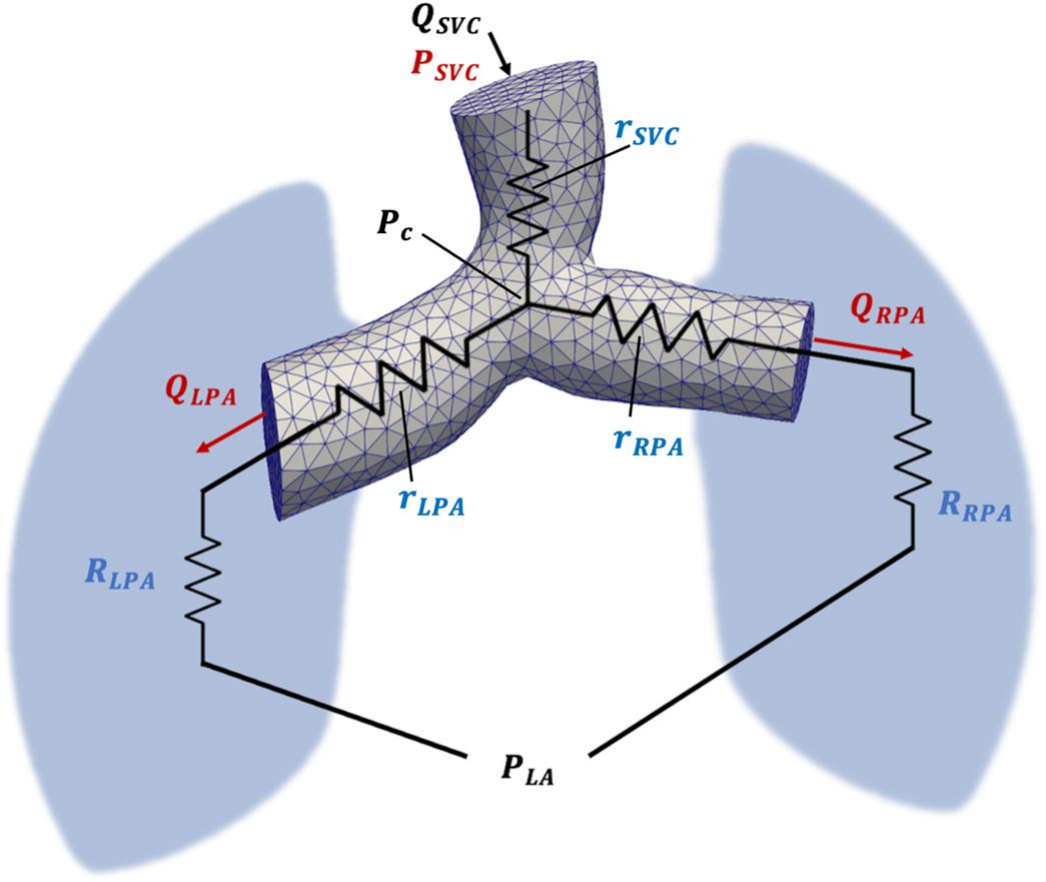
Schematic representation of the computational model used for PVR estimation using CFD. Inlet ($Q_{SVC}$) and outlet ($Q_{LPA}$, $Q_{RPA}$) flow is acquired from CMR flow measurements, while SVC, LPA, RPA and LA pressure is acquired from Cath pressure measurements. The pressure at the confluence of the SVC, LPA, and RPA is denoted by P_c_ and was calculated from CFD simulation results. Proximal SVC, LPA, and RPA resistances are denoted as $r_{SVC}$, $r_{LPA}$, and $r_{RPA}$, respectively.

The proximal resistances are computed from time-averaged flow rates and pressure gradients derived from the CFD simulation results (Figure 1). The pressure gradients are determined using the average pressure at a point near the center of the confluence of the SVC, LPA, and RPA, denoted as $P_{c}$. A detailed description of the PVR estimation algorithm is provided in (14). The proximal resistances can be combined with the distal resistances to account for the resistance along the entire LPA or RPA pathway:

(4) $${\tilde{R}}_{LPA}=R_{LPA}+r_{LPA},{\tilde{R}}_{RPA}=R_{RPA}+r_{RPA}.$$

These combined individual lung resistances can be used in Equation 3 to calculate the total PVR (${\tilde{R}}_{total}$), which is analogous to the value obtained via the Fick principle.

### 2.3 Statistical analysis

Descriptive statistics for all three centers are presented as mean ± standard deviation. Normality of the data was assessed using the Shapiro–Wilk test (18). To compare PVR estimates across the three centers, the Kruskal–Wallis rank sum test was employed (19). Paired data were analyzed using Wilcoxon rank sum tests (20), intraclass correlation coefficients (ICC) (21) with 95% confidence intervals (CI), and Bland–Altman analysis (22). A *p*-value of < 0.05 was considered statistically significant.

## 3 Results

### 3.1 Flow and pressure data

Comparison of clinically measured flow values across the three centers using the Kruskal–Wallis test revealed no statistically significant differences in flow within the SVC, LPA, or RPA (Table 1). In contrast, analysis of pressure measurements obtained via Cath showed significant differences among centers, with Center 1 exhibiting lower mean pressures in the SVC, LPA, RPA, and LA. However, despite these lower absolute pressures in Center 1, the mean pressure gradients in both the LPA ($P_{LPA}-P_{LA}$) and RPA ($P_{RPA}-P_{LA}$), did not differ significantly between centers.

**Table 1:** Clinically measured mean flow and pressure values across three centers.

Center	*N*	Q_SVC_ (L/min)	Q_LPA_ (L/min)	Q_RPA_ (L/min)	P_SVC_ (mmHg)	P_LPA_ (mmHg)	P_RPA_ (mmHg)	P_LA_ (mmHg)	*Δ*P_L_ (P_LPA_—P_LA_)	*Δ*P_R_ (P_RPA_—P_LA_)
1	16	1.07 ± 0.26	0.52 ± 0.20	0.56 ± 0.17	9.31 ± 1.49	8.88 ± 1.54	9.00 ± 1.55	4.44 ± 0.96	4.44 ± 1.26	4.56 ± 1.26
2	20	1.11 ± 0.33	0.47 ± 0.14	0.67 ± 0.25	12.30 ± 1.34	11.45 ± 1.28	11.85 ± 1.31	7.65 ± 1.53	3.80 ± 1.06	4.20 ± 1.11
3	6	1.00 ± 0.16	0.49 ± 0.14	0.51 ± 0.10	11.83 ± 2.14	10.83 ± 2.48	11.33 ± 2.42	7.83 ± 2.14	3.00 ± 1.67	3.50 ± 1.76
*P*-value	–	0.854	0.820	0.100	<0.001	<0.001	<0.001	<0.001	0.066	0.333

A nonparametric Kruskal–Wallis test revealed a significant difference in pressure measurements, with the mean pressures for Center 1 being considerably lower than for Centers 2 and 3. However, no significant difference in left/right pressure gradients and flow measurements was observed across the three centers.

### 3.2 Fick-based PVR vs. Cath-CMR-based PVR

**Figure 2:**
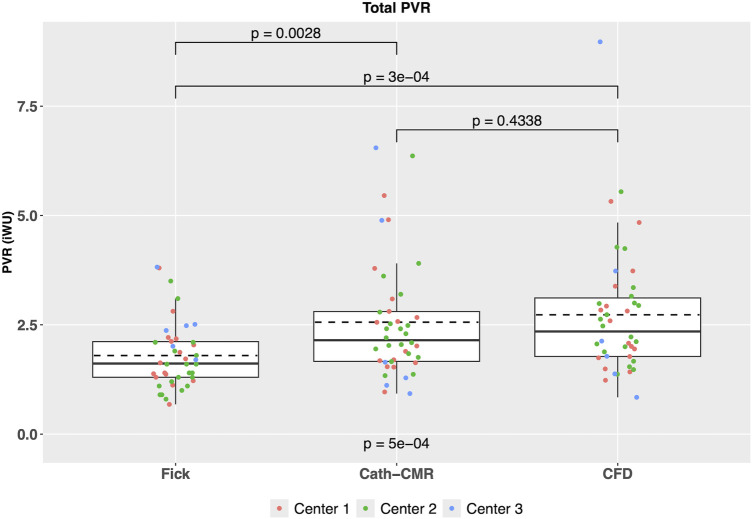
Comparison of total PVR between the three PVR estimation methods across all three centers indicates a significant difference between the Fick-based PVR and both the Cath-CMR and CFD-based PVR. In contrast, no statistical difference was found between the Cath-CMR-based PVR and CFD-based PVR.

As shown in Figure 2, total PVR estimated using the Fick-based method showed a significant difference compared to the PVR using the Cath-CMR method (Fick: 1.80 ± 0.77 iWU; Cath-CMR: 2.56 ± 1.36 iWU). On average, the Fick-based method produced PVR values approximately 0.75 iWU lower than those obtained using the Cath-CMR method. Overall, the two methods demonstrated poor agreement with an ICC of 0.22 [*p* = 0.055; 95% CI: (−0.05, 0.47)].

### 3.3 CFD-based PVR estimation and comparison to Fick and Cath-CMR methods

**Figure 3:**
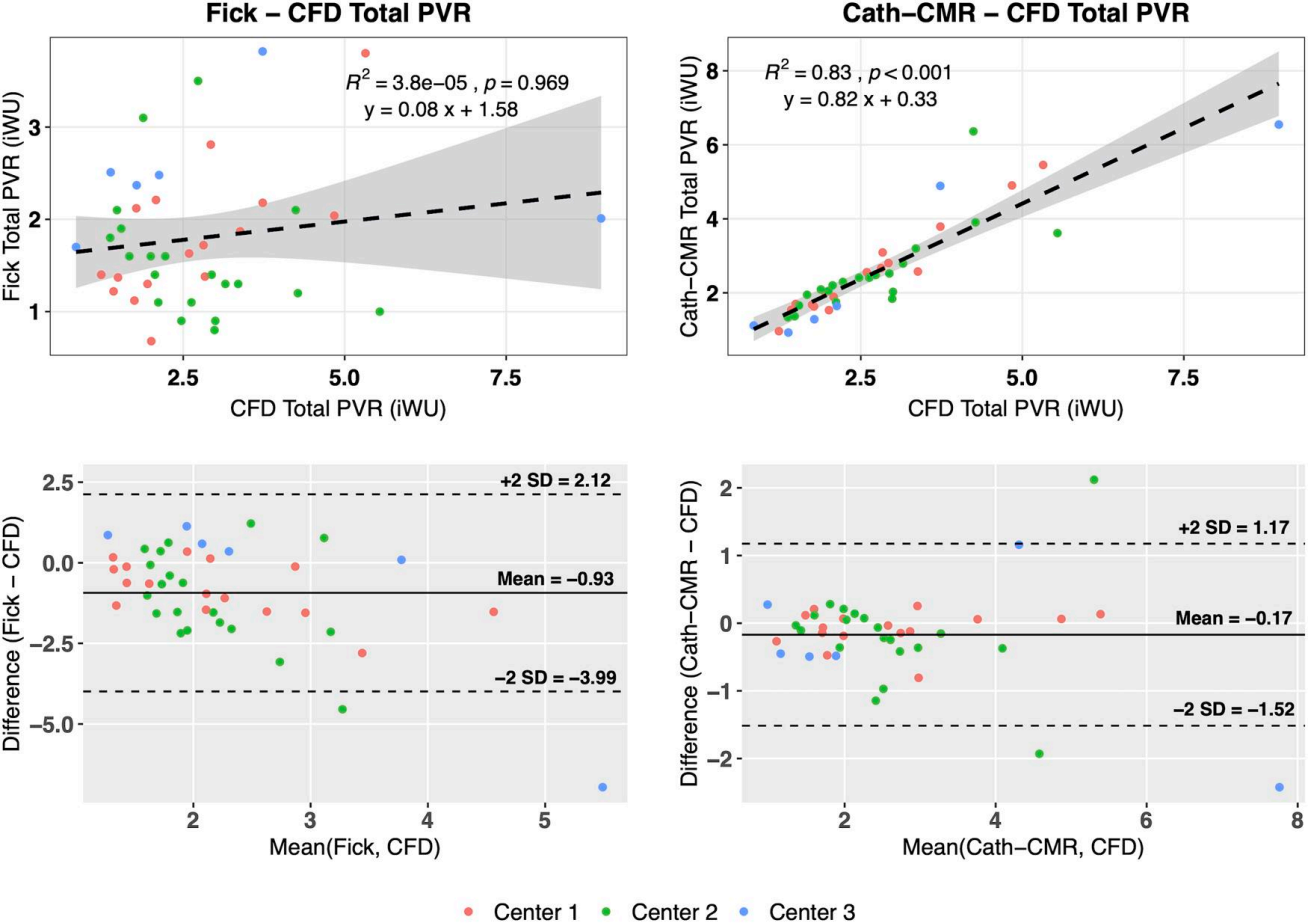
The Fick-based total PVR estimates show poor correlation with those of the CFD-based method, with the Fick-based method being different, on average, by −0.93 iWU. The Cath-CMR PVR, however, shows good correlation with the CFD-based PVR, with only a slight difference of −0.17 iWU.

For all three centers, individual left and right lung PVRs were computed using the CFD-based optimization algorithm. The resulting mean PVR was ${\tilde{R}}_{LPA}=\;$ 6.28 ± 3.19 iWU for the left lung and ${\tilde{R}}_{RPA}=$ 5.31 ± 3.74 iWU for the right lung, which includes the proximal resistances $r_{LPA}=\;$ 1.06 ± 1.26 iWU and $r_{RPA}=\;$ 0.66 ± 0.69 iWU for the left and right lung, respectively. Using Equations 3 and 4, the total PVR ${\tilde{R}}_{total}$ was computed from ${\tilde{R}}_{LPA}$ and ${\tilde{R}}_{RPA}$, yielding a mean ${\tilde{R}}_{total}=$ 2.73 ± 1.48 iWU. As shown in Figure 3, poor agreement was observed between the Fick-based method and the CFD-based method [ICC = 0.10; *p* = 0.22; 95% CI: (−0.13, 0.34)], with the Fick method underestimating the total PVR of the CFD method by 0.93 iWU on average. In contrast, the CFD method showed much stronger agreement to the Cath-CMR method [ICC = 0.88; *p* < 0.001; 95% CI: (0.79, 0.93)], with the Cath-CMR method underestimating the total PVR of the CFD method by only 0.17 iWU. The total PVR for all three methods and across all three centers is summarized in Table 2.

**Table 2:** Summary of mean total PVR estimates of the three methods.

Total PVR	Fick	Cath-CMR	CFD	*P*-value
Center 1	1.80 ± 0.75	2.55 ± 1.25	2.63 ± 1.20	0.057
Center 2	1.59 ± 0.70	2.51 ± 1.13	2.68 ± 1.07	<0.001
Center 3	2.48 ± 0.73	2.74 ± 2.38	3.14 ± 3.02	0.587
*P*-value	0.017	0.531	0.452	

**Figure 4:**
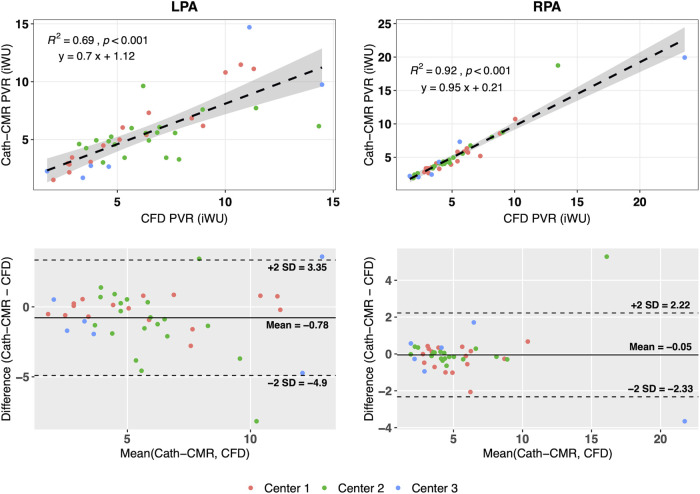
The individual lung resistances using the Cath-CMR method correlate well with those of the CFD method, particularly for the RPA. However, the Cath-CMR-based LPA PVR estimates show a bias of −0.78 iWU relative to the CFD-based LPA PVR, which is likely due to the increased proximal resistance in the LPA.

**Figure 5:**
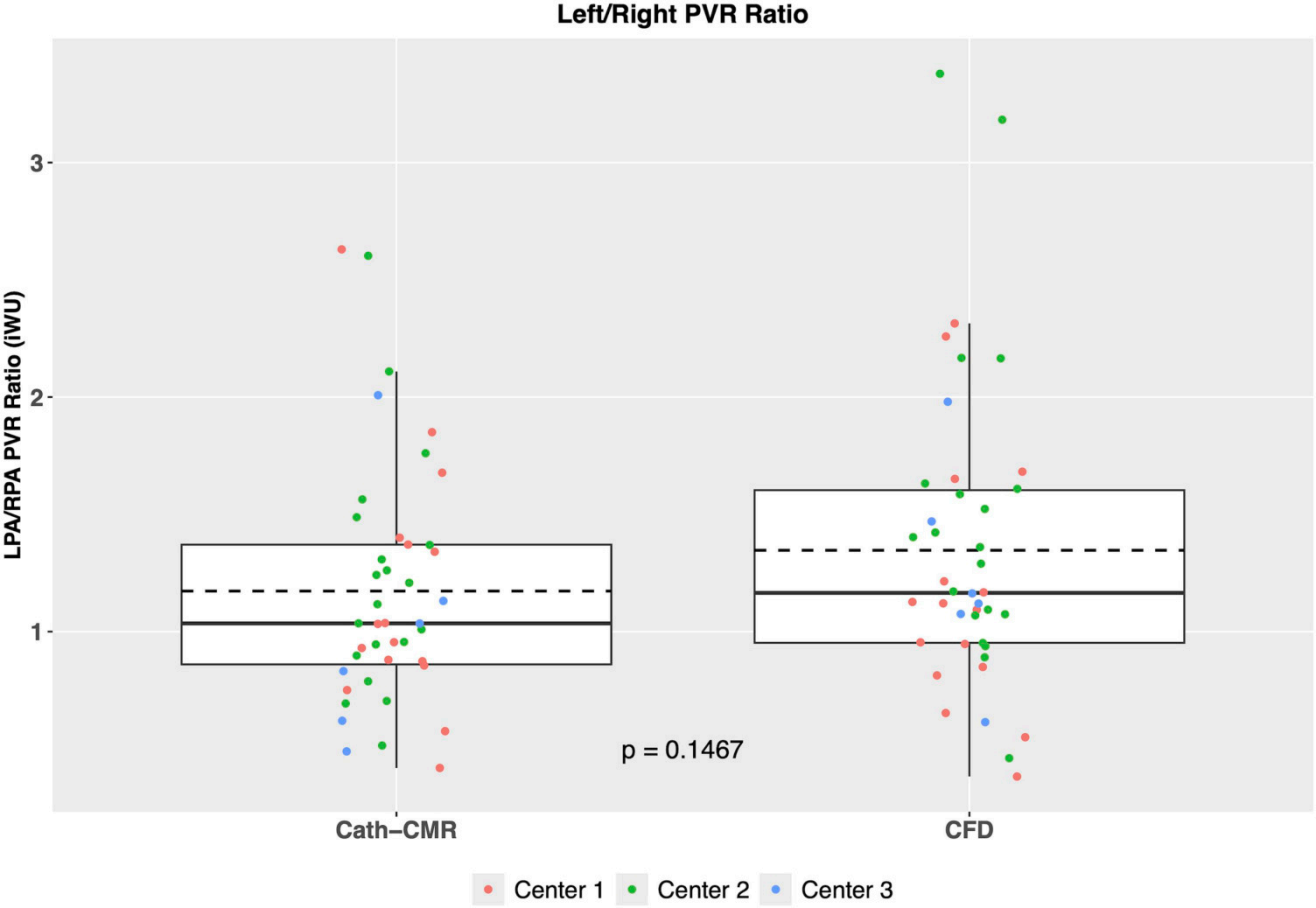
The ratio of LPA to RPA PVR reveals no significant difference between the Cath-CMR and CFD methods. Both methods demonstrate a slightly higher mean LPA resistance compared to RPA resistance.

The mean individual PVRs (including proximal resistances) across all three centers using the CFD method were 6.28 ± 3.19 iWU for the left lung and 5.31 ± 3.74 iWU for the right lung. In comparison, the Cath-CMR method produced mean individual PVRs of 5.50 ± 2.91 iWU for the left lung and 5.26 ± 3.74 iWU for the right lung. It should be noted that the LPA resistance shows a larger discrepancy between the two methods, which can be attributed in part to the proximal resistances found in the CFD method, which were larger for the LPA (1.06 ± 1.26 iWU) than for the RPA (0.66 ± 0.69 iWU). Still, the two methods’ individual PVR estimates show good agreement, with an ICC of 0.74 [*p* < 0.001; 95% CI: (0.56, 0.86)] for the left lung and an ICC of 0.95 [*p* < 0.001; 95% CI: (0.91, 0.97)] for the right lung. Similarly, as shown in Figure 4, the two methods show good correlation, particularly in the RPA, with the Cath-CMR-based PVR estimates underestimating the CFD-based PVR estimates by an average of 0.78 iWU for the left lung and 0.05 iWU for the right lung. The ratio of LPA and RPA resistance also indicates no significant difference between the individual lung PVR estimates of the Cath-CMR method and the CFD method, with a mean LPA/RPA ratio of 1.17 ± 0.52 for the Cath-CMR method and 1.35 ± 0.64 for the CFD method, as shown in Figure 5.

## 4 Discussion

**Figure 6:**
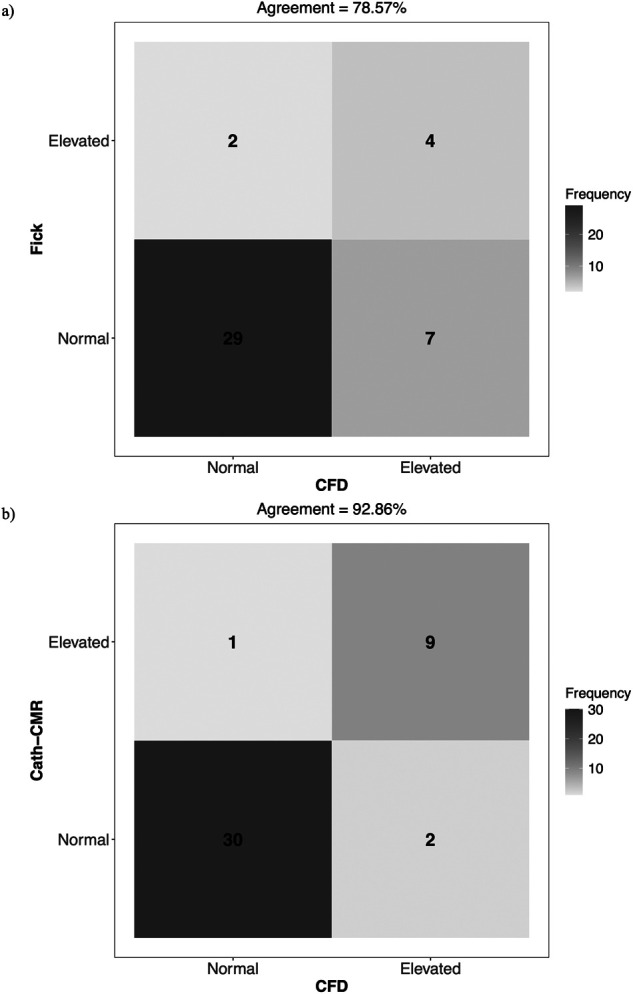
Confusion matrix of Fontan failure risk stratification agreement for **(a)** Fick vs. CFD and **(b)** Cath-CMR vs. CFD, using PHVD as an indicator. In this context, “Normal” refers to a transpulmonary pressure gradient ≤ 6 mmHg and an indexed PVR ≤ 3 iWU, while “Elevated” indicates values exceeding one of these two thresholds.

This three-center study on PVR estimation in children with Glenn vasculature reveals two important findings. First, there are substantial discrepancies in total PVR estimates between the Fick-based method and both the Cath-CMR and CFD methods. Several factors may contribute to the discrepancies in total PVR. One source of possible error is in the calculation of the transpulmonary pressure gradient which, as seen in Equation 1, is computed as the difference in mPAP and PAWP, as measured by Cath. The PAWP measurement as a surrogate to direct LA pressure measurement has been shown to significantly overestimate LA pressure in pre-Fontan patients, resulting in an underestimation of PVR (23). Another source of possible error lies in the limited interchangeability between pulmonary blood flow measured via the Fick principle and that measured by CMR, which has been shown in previous single-center studies (12, 13, 24, 25). One reason for this is the $VO_{2}$ measurement in the calculation of $Q_{p}$ in Equation 1, which can be particularly inaccurate for assumed $VO_{2}$ estimates using formulas or tables based on age or weight (12). The discrepancies in PVR estimates between the Fick method and the other two methods are potentially significant because the Fontan risk stratification, which is based in part on PVR values, relies on these estimates. For instance, pulmonary hypertensive vascular disease (PHVD) serves as an indicator of Fontan failure. The threshold for PHVD (and an elevated risk of Fontan failure) is a mean transpulmonary pressure gradient exceeding 6 mmHg or an indexed PVR surpassing 3 iWU for single ventricle physiology (26). Using this threshold, the Fick-based method classifies 36 cases as normal risk, while the CFD method classifies only 29 as normal, as illustrated in Figure 6. Of the remaining six cases which were classified as elevated risk by the Fick method, only four were also classified as such by the CFD method. Overall, the Fick-based method demonstrated an agreement of 78.6% with the CFD method in terms of PHVD risk classification. In contrast, the Cath-CMR method demonstrated a classification agreement of 92.9% with the CFD method. However, even a 7% PHVD misclassification poses a risk of performing Fontan surgery on high-risk cases that may not be suitable for the procedure. For instance, misclassifying a patient as low-risk and proceeding with the Fontan procedure could lead to complications such as high central venous pressure, low cardiac output, or pleural effusion. Conversely, erroneously classifying a patient as high-risk may unnecessarily delay the Fontan procedure to start a new medication.

The second finding is that the proximal resistances included in the CFD method significantly contribute to the PVR, particularly for the LPA. As shown in Figure 4, the correlation between Cath-CMR-based LPA PVR and CFD-based PVR is significantly lower than for the RPA, along with a much higher bias. This discrepancy is likely attributed to the increased proximal resistance exhibited by the CFD method in the LPA (1.06 ± 1.26 iWU), which is significantly greater than that of the RPA (0.66 ± 0.69 iWU). The increased LPA proximal resistance is understandable, as the LPA outlet surface area in the CFD models is on average smaller than that of the RPA, with a mean of 0.50 ± 0.39 cm^2^ for the LPA and 0.58 ± 0.32 cm^2^ (*p* = 0.069). This is attributable to anatomical factors such as the large Damus-Kaye-Stansel anastomosis performed during the Norwood operation, which often rests on the proximal LPA, exerting external compression and narrowing the vessel. Another possible factor contributing to the discrepancy in proximal resistance between the LPA and RPA is their length. In our future research, we plan to investigate the impact of pulmonary artery length on proximal resistance. However, we anticipate that this effect will be less pronounced compared to the influence of the pulmonary arterial radius. This is because the models of pulmonary arteries are roughly cylindrical in shape, which means that resistance increases linearly with vessel length while it exhibits an inverse fourth-order relationship with the pulmonary arterial radius.

Overall, the CFD method for estimating PVR may be advantageous to both the Fick and Cath-CMR methods. First, it allows the determination of individual lung resistances, something that is not typically done with the Fick method. Second, it does not rely on $Q_{p}$ measurements derived from $VO_{2}$ measurements and arteriovenous oxygen content. Instead, it uses $Q_{p}$ measured by CMR, which is the gold standard for pulmonary blood flow measurement. Finally, the CFD method offers a more complete representation of pulmonary resistances compared to the Cath-CMR method, as it incorporates both distal and proximal resistances in the LPA and RPA.

However, several limitations must be acknowledged. First, the CFD PVR estimation algorithm has only been tested using a simplified model of the Glenn vasculature. It does not currently account for vessel wall compliance or variations in blood viscosity, both of which may influence resistance and should be explored in future work using more realistic models. Including compliance in the CFD model would allow for a more accurate optimization to the clinical flow curves by adding an additional parameter for exploration. However, the introduction of an additional optimization parameter may lead to issues in identifiability (27, 28), which warrants further exploration alongside model compliance in future studies. An alternative approach to conducting an identifiability analysis is to approximate compliance directly from distensibility (29), thereby avoiding potential issues of identifiability associated with the introduction of compliance. Incorporating compliance may also enable more precise pressure wave dynamics, which may affect the transpulmonary pressure gradient used in the CFD optimization (30). Blood viscosity is generally assumed to be Newtonian in large vessels such as the pulmonary arteries (31), so it is not expected to impact PVR significantly. However, if the model was extended further into the lungs where vessel size decreases significantly, blood viscosity should be modeled as non-Newtonian.

Second, the sample size was limited to fewer than 50 patients. A larger cohort would enhance the statistical power and generalizability of the findings, which is particularly important for determining the concordance between Fontan risk classifications and PVR estimation methods. An increased sample size would also improve the reliability of ICC estimates and allow for more meaningful subgroup analyses.

Third, the timing between Cath and CMR exams varied among patients. Some underwent both procedures on the same day, while others had them separated by up to a year, potentially introducing temporal variability in the measurements. However, since most patients had their Cath and CMR exams on the same day, no significant differences in the statistical conclusions are expected. As the sample size grows in future studies, it would be intriguing to observe the impact of temporal misalignment between Cath and CMR exams.

Finally, the Cath-CMR and CFD methods neglected to consider collateral flow. However, it is anticipated that the presence of collateral flow would have a similar impact on the Fick, Cath-CMR, and CFD methods, causing them to underestimate the total pulmonary flow and consequently leading to an increased PVR. Given these limitations, further clinical study is needed to determine which PVR method most effectively correlates with clinical outcomes.

## 5 Conclusion

The comparison of methods for computing PVR revealed inconsistencies between the Fick-based method and both the Cath-CMR and CFD methods in patients with Glenn physiology, as observed across three centers. The CFD-based computational optimization method for PVR estimation demonstrated excellent agreement with the Cath-CMR method for RPA PVR estimation, but only modest agreement in LPA PVR estimation. This underscores the significance of considering both proximal and distal lung resistance, potentially making the CFD method more suitable than the Cath-CMR method. Further research is warranted to determine which method represents the most accurate quantification of PVR in pre-Fontan patients.

## Data Availability

The data analyzed in this study is subject to the following licenses/restrictions: Data cannot be shared publicly because of the policies of Children's Hospital Colorado, Children's National Hospital, and West Virginia University Medicine Children's Hospital. Data are available from the respective ethics committees for researchers who meet the criteria for access to confidential data. Requests to access these datasets should be directed to; Heather Byrd, heather.byrd@cuanschutz.edu; Yue-Hin Loke, YLoke@childrensnational.org; Candie DaFonzo, candace.dafonzo@wvumedicine.org.
